# Association between anlotinib trough plasma concentration and treatment outcomes in advanced non-small-cell lung cancer

**DOI:** 10.3389/fonc.2023.1146362

**Published:** 2023-03-03

**Authors:** Ling Chen, Hong Jiang, Jun-jie Rao, Liu-sheng Wang, Wei Yan, Jian Ye, Jiang Lou

**Affiliations:** ^1^ Department of Pharmacy, Affiliated Hangzhou First People’s Hospital, Zhejiang University School of Medicine, Hangzhou, China; ^2^ Department of Clinical Pharmacy, Key Laboratory of Clinical Cancer Pharmacology and Toxicology Research of Zhejiang Province, Affiliated Hangzhou First People’s Hospital, Zhejiang University School of Medicine, Hangzhou, China; ^3^ Department of Cardiothoracic Surgery, Affiliated Hangzhou First People’s Hospital, Zhejiang University School of Medicine, Hangzhou, China; ^4^ Department of Clinical Laboratory, Affiliated Hangzhou First People’s Hospital, Zhejiang University School of Medicine, Hangzhou, China; ^5^ Department of Respiratory, Affiliated Hangzhou First People’s Hospital, Zhejiang University School of Medicine, Hangzhou, China; ^6^ Department of Respiratory, Zhejiang Hospital, Hangzhou, China

**Keywords:** anlotinib, trough plasma concentration, clinical efficacy, toxicities, non-small cell lung cancer

## Abstract

**Background:**

Efficacy and toxicities of anlotinib (ANL) show large inter-patient variation, which may partly be explained by differences in ANL exposure. Exposure-response/toxicities relationship have not been investigated for ANL. Therefore, the aim of the present study was to explore the association between the trough plasma concentration (C_trough_) of ANL and treatment outcomes in Chinese patients with advanced non-small cell lung cancer (NSCLC).

**Methods:**

Patients with advanced NSCLC who started third-line or further ANL alone therapy between January 2021 and October 2022. This study examined the ANL C_trough_ and clinical response evaluation at day 43 after initiation of ANL treatment. We evaluated the association between the ANL C_trough_ and clinical efficacy and toxicities. Additionally, this study defined patients with complete response (CR), partial response (PR) and stable disease (SD) as responder. The receiver-operating characteristic (ROC) curve combined with Youden index was identify the potential threshold value of ANL C_trough_ for the responder.

**Results:**

52 patients were evaluated for analyses. The median ANL C_trough_ was 11.45ng/ml (range, 3.69-26.36 ng/ml). The ANL C_trough_ values in the PR group (n=6, 15.51 ng/ml (range, 8.19-17.37 ng/ml)) was significantly higher than in the PD group (n=8, 7.44 ng/ml (range, 5.41-14.69 ng/ml), *p*=0.001). The area under the ROC curve (AUC_ROC_) was 0.76 (95% confidence interval (CI), 0.58-0.93; *p*=0.022) and threshold value of ANL C_trough_ predicting responder was 10.29 ng/ml (sensitivity 65.9% and specificity 87.5%, the best Youden index was 0.53). The disease control rate (DCR) was 84.6%, and DCR was significantly higher in the high-exposure group (≥10.29ng/ml) than low-exposure group (<10.29ng/ml) (96.67% vs 68.18%, *p*=0.005). Although there was no significant difference in ANL C_trough_ between grade ≥ 3 and grade ≤2 toxicities, the incidence of any grade hand-foot syndrome (70.0% vs 36.36%, *p*=0.016) and thyroid-stimulating hormone elevation (53.33% vs 22.73%, *p* =0.026) was significantly higher in the high-exposure group compared with the low-exposure group.

**Conclusions:**

Considering these results, we propose that maintaining ANL C_trough_ ≥ 10.29ng/ml was important for achieving the response in advanced NSCLC patients treated with ANL.

## Introduction

Anlotinib (ANL) is a multi-targeted receptor tyrosine kinase inhibitor (TKI), which was developed by Jiangsu Chia-Tai Tianqing Pharmaceutical Group Co., Ltd. The major targets of ANL including vascular endothelial growth factor receptors (VEGF) 2 and 3, fibroblast growth factor receptors 1 to 4, platelet-derived growth factor receptors, c-Kit and Ret, resulting in treatment of various tumors (1, 2). ANL was approved as a third-line or further treatment for refractory advanced non-small cell lung cancer (NSCLC) by the National Medical Products Administration (NMPA) of China in 2018 (3). In phase 3 clinical study (ALTER 0303), ANL can significantly prolong overall survival (OS) and progression-free survival (PFS) and improve the quality of life in advanced NSCLC (4). The common toxicities during the treatment of NSCLC were hypertension, gastrointestinal reaction, hand-foot syndrome, and so on (4). Toxicities of grade 3 or higher were reported 21.67-61.9% and some patients led to dose reduction or treatment interruption (5; Han and Li and 6). Therefore, the optimal dose adjustment of ANL is crucial for avoiding serious toxicities and maximize its clinical efficacy.

As with other TKIs, the dose regimen of ANL are fixed and was adjusted mainly according to the degree of toxicities, the treatment regimen is 8mg, 10mg, or 12mg daily dose for 2 weeks on treatment followed by 1 week off treatment. In order to improve clinical efficacy and reduce toxicities, therapeutic drug monitoring (TDM) of other TKIs has been attempted for optimal dosage. For example, the sum of the total trough plasma concentration of both sunitinib and its active metabolite SU12662 should be set above 50ng/ml to obtain clinical efficacy and below 100ng/ml to avoid serious toxicities in sunitinib treated with metastatic renal cell carcinoma (7). In multiple studies, TDM may be useful for reducing toxicities and increasing clinical efficacy of imatinib, sunitinib and pazopanib (7). According to the phase I clinical trial, pharmacokinetic (PK) show that ANL is rapidly absorbed, high protein binding rates (93%) and eliminated slowly with a half-life (t_1/2_) of 96h, the trough plasma concentration (C_trough_) of ANL was reached at day 22 before the next treatment cycle was 5.05-28.5ng/ml (8). *Zhang et al.* have been reported that the median ANL C_trough_ was 25.38ng/ml (range 11.61-62.9ng/ml), which was characterized by high inter-patient variability in PK with 47.5% coefficients of variation (CV) (9). Several reports have also shown that ANL PK are partially dose-dependent and large inter-patient variability between 39.83% and 79.36%, which was similar to the other TKIs, such as imatinib, pazopanib and sunitinib (10, 11). ANL is mainly metabolized by CYP1A2 and CYP3A4/5 and secondary metabolic enzymes including CYP2C9, CYP2C19 and CYP2D6 (12, 13). Dexamethasone is a moderate inducer of CYP3A, which could accelerate the metabolism of ANL and reduce exposure (14). CYP2C19 rs3814637 and rs11568732 gene polymorphisms may also influence the ANL exposure (15). In addition, *Li et al.* and *Yu et al.* has been reported that ANL plasma concentration is higher in female patients than male patients under the same dose (14, 16). Beside these known contributions to variability in ANL exposure, patient characteristics such as age, body mass index, renal function and liver function may also contribute to variability in ANL exposure. Although those factors did not affect exposure separately, a relevant cumulative effect on plasma exposure cannot be excluded. Hence, TDM could be an effective tool for dose optimization of ANL according to plasma concentration. However, the information on the association between ANL exposure and treatment outcomes is limited.

In the present study, we aimed to evaluate the association of ANL C_trough_ with clinical efficacy and toxicities in Chinese patients with advanced NSCLC.

## Material and methods

### Patients

This prospectively study was performed in advanced NSCLC patient treatment with ANL alone at Hangzhou First People’s Hospital between January 2021 and October 2022. All patients were disease progression after at least 2 lines of chemotherapy combined with or without immune check point inhibitors for patients without driver alterations as well as disease progression after at least 1 line of chemotherapy combined with or without immune check point inhibitors and TKI therapy for patients with driver alterations. The dosage regimen of ANL is 8mg, 10mg, or 12mg daily dose for 2 weeks on treatment followed by 1 week off treatment of a 21-day cycle. Daily dosing was determined individually by each attending doctor based on the patient’s age, bodyweight, Eastern Cooperative Oncology Group (ECOG) Performance Status (PS) and so on. Patients were evaluated for clinical efficacy and plasma concentration on day 43 after initiation of ANL treatment. This study was approved by the Ethics Committee of the Hangzhou First People’s Hospital (Ethical approval documents: No. 2020-098-01) and the registration number ChiCTR2100043709 was granted by the Chinese Clinical Trial Registry, and written informed consent was obtain from all patients. We extracted clinical data including age, sex, bodyweight, dosage, metastasis, etiology, TNM stage, PS score from the electronic medical records.

### Measurement of ANL C_trough_

Previous studies have reported that ANL plasma concentration were measured using ultra-high-performance liquid chromatography coupled to tandem mass spectrometry (UPLC-MS/MS) (10, 17, 18). However, we were unable to obtain the internal standard in the literature. Trough methodological research, this study found that zanubrutinib is a suitable new internal standard. A new specific and sensitive UPLC-MS/MS has been developed and application for determination of ANL C_trough_ in this study. Briefly, plasma sample were treated by protein precipitation and addition of zanubrutinib as internal standard, then 5.0μL of the supernatant was analyzed and separated on an Ultimate XB-C18 column (100mm × 2.1mm, 3.0μm particle size) with optimized gradient of mobile phase A (consisted of 10mM ammonium acetate-0.1% formic acid) and mobile phase B (acetonitrile) was used to elute ANL, the gradient elution procedure was set as follows: 0-2.5min, 10%B→50%B; 2.5-2.6min, 50%B→100%B; 2.6-3.5min, 100%B; 3.5-5.0min, 100%B→10%B. The Mass spectrometric detection was performed on a Xevo TQD Triple Quadrupole Mass Spectrometry (Waters, USA) with an electrospray ionization source coupled with the above described UPLC system. Quantitative analysis was conducted using MRM transitions of m/z 408.1→339.1 for ANL and 472.2→290.1 for zanubrutinib, respectively. The calibration curve of ANL in plasma was linear over the concentration range of 1.0-100.0ng/mL with a correlation coefficient r^2^>0.990. The precision and accuracy was less than 8.64% and within ±104.61%, respectively. The extraction recovery and matrix effect was 104.81-107.32% and 102.54%-104.26%, respectively. The method was validated and accorded with the criteria of industrial guidance for bioanalytical method validation of the Food and Drug Administration. (FDA. 2018. Guidance for industry, bioanalytical method validation. FDA, Washington, DC.) Blood samples (4ml aliquots) were collected from 52 patients on day 43 after the initiation of ANL treatment to measure ANL C_trough_. Patients could not be evaluated for ANL C_trough_ were excluded.

### Assessment of efficacy and safety

Efficacy evaluation was assessed in accordance with the Response Evaluation Criteria in Solid Tumors (RECIST) guidelines, version 1.1, which were included complete response (CR), partial response (PR), stable disease (SD), or progressive disease (PD) based on the computed tomography or magnetic resonance imaging. The assessment of clinical efficacy was performed at day 43 after the initiation of ANL treatment. According to the *Noda S et al.* study, we defined patients with CR, PR and SD as responder, and defined patients with PD as non-responder in this study (19). Disease control rate (DCR) was defined as the percentage of patients with CR, PR and SD. Patients could not be evaluated for efficacy were excluded. Toxicities were registered from the start of treatment until at day 43 according to the Common Terminology Criteria for Adverse Events (CTCAE) version 5.0. Toxicities of worst grade were recorded within first two cycles. Additionally, we examined association between the median ANL C_trough_ and clinical efficacy and toxicities.

### Statistics analysis

Categorical variables statistically analyzed with the Chi-square test or Fisher’s exact test, and continuous variables were statistically analyzed using the Mann-Whitney U-test, significance was set at *p*<0.05. Kruskal-Wallis H with Bonferroni-corrected test was performed for the three-group comparison. The adjusted *p* values by Bonferroni-corrected test multiplied the *p* values of each paired comparison group by 3, and adjusted *p* values of < 0.017 were considered statistically significant for the comparison between the two groups. Patients were divided according to the PR, SD and PD, different dosing doses of ANL and toxicities (grade ≥3 or graded ≤2 toxicities). Comparing the ANL C_trough_ between PR, SD and PD, and different dosing doses of ANL using Kruskal-Wallis H with Bonferroni-corrected test. Comparing the toxicities grade ≥3 and graded ≤2 toxicities with Mann-Whitney U-test. The sensitivity and specificity of the analyzed data were calculated by receiver operating characteristic (ROC) curves, and predictive capacity is expressed by the area under the curve (AUC) value. The optimal ANL C_trough_ for prediction of responder was determined by Youden index (sensitivity+specificity-1). Patients also were divided into high-exposure group (above prediction concentration) and low-exposure group (below prediction concentration) according to the prediction concentration. Patient characteristics and the incidence of any grade toxicities and grade ≥ 3 toxicities were compared between low-exposure group and high-exposure group. Univariate and multivariate logistic regression analyses were evaluated to potential factors associated with DCR. Variables with borderline significance (*p*<0.2) on the univariate analysis were subjected to multivariate logistic regression analyses, *p*<0.05 were considered statistically significance in the multivariate analysis (20). All statistical analyses were performed using SPSS 22.0.

## Results

### Patient characteristics

**Figure 1:**
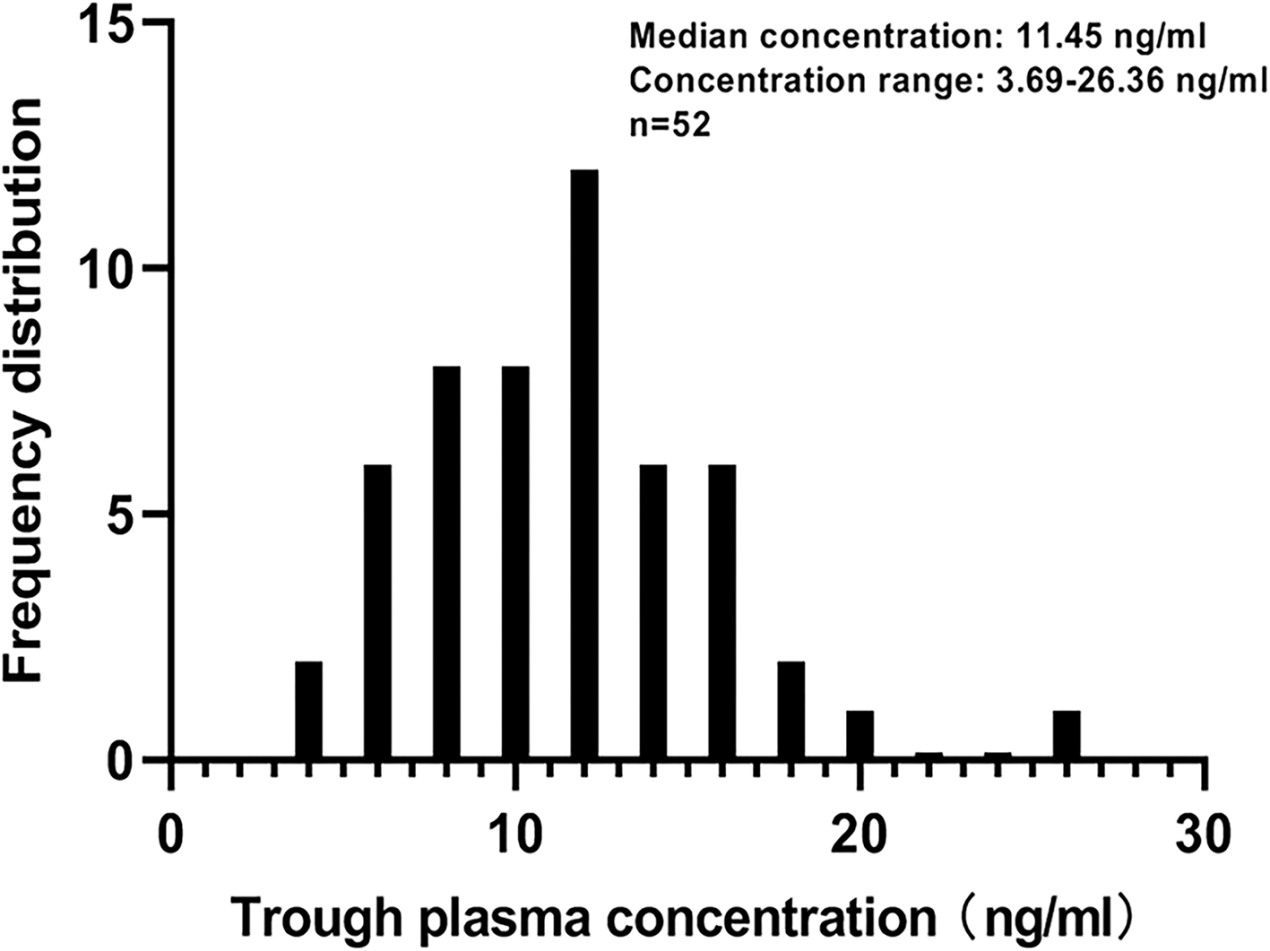
Distribution of anlotinib trough plasma concentration.

58 patients treated with ANL were enrolled in this study. We excluded one patient for whom treatment was discontinued within 43 days owing to grade 3 hand-foot syndrome toxicity. Two patients who were rapid clinical deterioration and could not be evaluated for ANL C_trough_ at day 43 after the initiation of ANL treatment. Three patients could not be evaluated for efficacy who were changed hospitals. The above-mentioned six patients were excluded from analysis. 52 patients were included and baseline characteristics are shown in **Table 1**. The median age was 66 years (range, 44-84 years) and median body weight was 60 kg (range, 41-89 kg). The dosage regimen of ANL was 12mg in 15 patients, 10mg in 24 patients, and 8mg in 13 patients. ANL C_trough_ were higher than LLOQ (1.0 ng/ml) from 52 patients. The median (minimum-maximum) and mean ± SD plasma concentrations of ANL C_trough_ was 11.45ng/ml (range, 3.69-26.36 ng/ml) and 11.38 ± 4.29ng/ml (n=52, CV=37.66%), which shown large inter-patient variability in ANL C_trough._ The distribution of ANL C_trough_ were presented in **Figure 1**.

**Table 1:** Baseline characteristics of patients.

Characteristic	Value (median, range) or (%)
Age(years)	66 (44-84)
Body weight (kg, range)	60 (41-89)
Body surface area (m^2^, range)	1.61 (1.38-2.01)
Dosage	
12mg	15 (28.85%)
10mg	24 (46.15%)
8mg	13 (25.00%)
Gender, n (%)	
Male	31 (59.62%)
Female	21 (40.38%)
Smoking history, n (%)	
Never	29 (55.76%)
Current/former	23 (44.23%)
ECOG PS, n (%)	
0	12 (23.08%)
1	31 (59.62%)
2	9 (17.30%)
Histology, n (%)	
Adenocarcinoma	31 (59.62%)
Squamous	21 (40.38%)
TNM stage, n (%)	
III stage	18 (34.62%)
IV stage	34 (65.38%)
Driver alterations	
No	37 (71.15%)
Yes	15 (28.85%)
EGFR mutation	10 (19.23%)
ALK rearrangement	2 (3.85%)
Other mutation	3 (5.77%)
Number of metastases	
≤3	21 (40.38%)
>3	31 (59.62%)
Efficacy of previous therapy	
Yes	48 (92.31%)
No	4 (7.69%)
Drugs of previous therapy	
Chemotherapy	8 (15.38%)
Chemotherapy+Immunotherapy	29 (55.77%)
Chemotherapy+Immunotherapy+Target	15 (28.85%)

ECOG, Eastern Cooperative Oncology Group; PS, performance status; TNM, tumor node metastasis.

### Association of efficacy with ANL C_trough_

**Figure 2:**
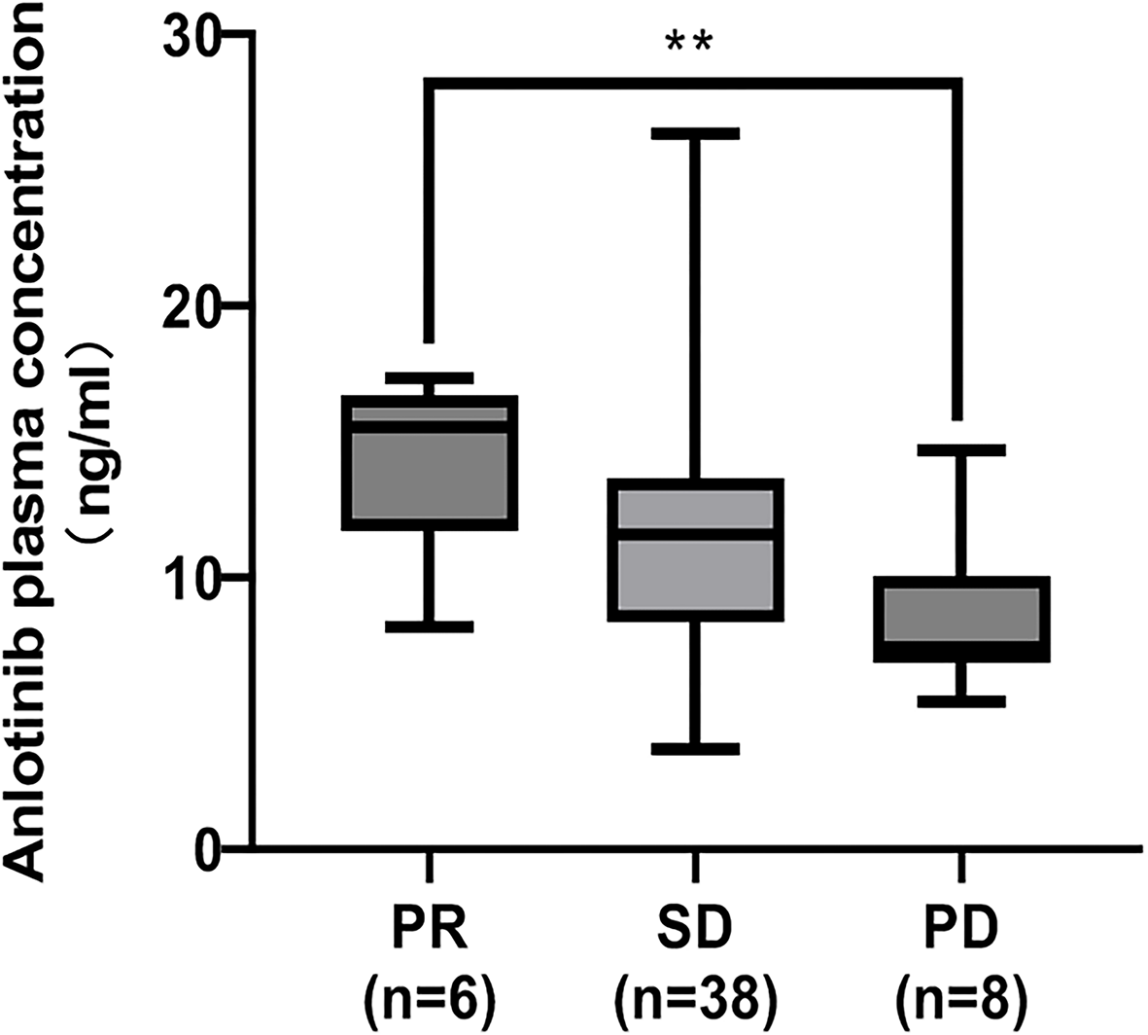
Comparison of the median ANL C_trough_ in patients with partial response (PR), stable disease (SD) and progressive disease (PD). The adjusted p values by Bonferroni-corrected test multiplied the p values of each paired comparison group by 3. **represents p<0.01.

**Figure 3:**
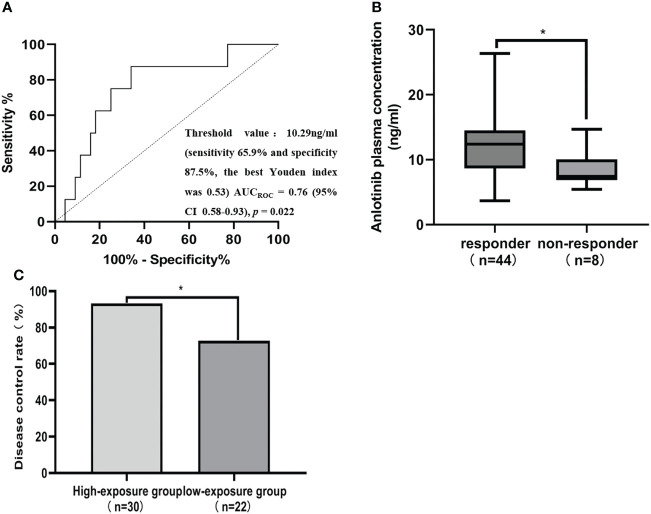
ANL C_trough_ threshold for responder and disease control rate (DCR). **(A)** Receiver operating characteristics (ROC) curves for predicting responder status. AUC_ROC_ area under the receiver operating characteristic curve, CI Confidence interval. **(B)** Comparison of ANL C_trough_ between patients with responder and non-responder. Responders were defined as the patients with CR and SD at best response, while non-responder were defined the patients with PD at best response. **(C)** Comparison of the DCR between patients with a median ANL C_trough_ ≥ 10.29 ng/ml (high-exposure group) and <10.29 ng/ml (low-exposure group), * represents p<0.05.

CR, PR, SD and PD were observed in 0, 6, 38 and 8 patients, respectively. Based on the results of plasma concentration, the median (minimum-maximum) and mean C_trough_ of ANL treatment with 8mg, 10mg and 12mg were 10.10ng/ml (range, 3.69-16.49) and 10.06ng/ml (CV=38.46%), 11.51ng/ml (range, 3.75-26.36ng/ml) and 11.42ng/ml (CV=41.52%), and 13.19ng/ml (range, 5.41-17.37 ng/ml) and 12.47ng/ml (CV=30.31%), respectively. Although there was high inter-individual variability among patients treated with the same dose of ANL, there was no significant correlation between the median ANL C_trough_ versus daily dose, which was shown in **Figure 1S**. The median C_trough_ of the PR (n=6), SD (n=38) and PD (n=8) groups were 15.51ng/ml (range, 8.19-17.37ng/ml), 11.56ng/ml (range, 3.69-26.36ng/ml) and 7.44 ng/ml (range, 5.41-14.69ng/ml), respectively, the result was presented in **Figure 2**. The median C_trough_ in the PR group was significantly higher than PD group (*p*=0.001), but median C_trough_ in the SD group was not significantly difference in the PD group (*p*=0.148), and median C_trough_ in the PR group was also not significantly difference in the SD group (*p*=0.181). The area under the ROC curve (AUC_ROC_) was 0.76 (95% confidence interval (CI), 0.58-0.93; *p*=0.022) and threshold value of ANL C_trough_ predicting responder was 10.29 ng/ml (sensitivity 65.9% and specificity 87.5%, the best Youden index was 0.53), which were depicted in **Figure 3**. Additionally, the median ANL C_trough_ was 12.39 ng/ml (range, 3.69-26.36ng/ml) in the responder group (n=44) was significantly higher than the non-responder group (n=8, 7.44ng/ml (range, 3.69-26.36ng/ml), *p*=0.022). We divided the patients into the high-exposure group (≥10.29ng/ml) and low-exposure group (<10.29ng/ml) according to the threshold value. DCR was significantly higher in the high-exposure group than in the low-exposure group (96.67% vs 68.18%, *p*=0.005).

The results of the comparison of patient characteristics in the high-exposure group and low-exposure group are shown in **Table 2**. ANL C_trough_ was significantly higher in the high-exposure group compared with the low-exposure group (11.90ng/ml vs 8.55ng/ml, *p*=0.021). There was significant difference in the dosage of administration between high-exposure group and low-exposure group (*p*=0.023). No significant difference was observed in the sex (*p*=0.613), age (*p*=0.290), body weight (*p*=0.242), body surface area (*p*=0.824), smoking history (*p*=0.473), ECOG PS (*p*=0.104), histology (*p*=0.613), driver alterations (*p*=0.404), number of metastases (*p*=0.281), TNM stage (*p*=0.414) and efficacy of previous therapy (*p*=0.466) among high-exposure group and low-exposure group.

**Table 2:** Comparison between patients with ANL C_trough_ ≥10.29ng/ml (high-exposure group) and <10.29ng/ml (low-exposure group).

Characteristic	high-exposure group (n=30)	low-exposure group (n=22)	*p* values
Age (years, range)	69 (48-81)	65 (44-84)	0.29
Body weight (kg, range)	58 (43-89)	60 (41-80)	0.242
Body surface area (m^2^, range)	1.61 (1.38-2.01)	1.66 (1.40-1.94)	0.824
Sex			
Male	17	13	0.613
Female	13	8	
Dosage			
≥10mg	26	13	**0.023**
8mg	4	9	
Smoking history			
Never	12	11	0.473
Current/former	18	11	
ECOG PS			
≤1	27	16	0.104
≥2	3	6	
Histology			
Adenocarcinoma	17	14	0.613
Squamous	13	8	
TNM stage			
III stage	9	9	0.414
IV stage	21	13	
Driver alterations			
No	20	17	0.404
Yes	10	5	
Number of metastases			
>3	14	7	0.281
≤3	16	15	
Efficacy of previous therapy			
Yes	27	21	0.466
No	3	1	
Drug Interactions			
Dexamethasone	2	0	0.217
Amlodipine	4	2	0.636
Nifedipine	3	2	0.913
losartan	1	3	0.168
Metformin	4	3	0.975
Insulin	3	1	0.466
Levothyroxine	2	4	0.199
Atorvastatin	6	3	0.549
Rosuvastatin	3	5	0.209

ECOG, Eastern Cooperative Oncology Group; PS, performance status; TNM, tumor node metastasis.

Bold values indicate that the difference were statistically significant.

### Factors associated with the DCR

**Table 3** illustrated the results of univariate and multivariate logistic regression analyses of the DCR in 52 patients. In the univariate analysis, ANL C_trough_ ≥10.29 ng/ml (*p*=0.020), age ≥65 years (*p*=0.133) and dosage ≥10mg (*p*=0.089) were below borderline significance (*p*<0.2). In the multivariate analysis, ANL C_trough_ ≥10.29 ng/ml was independently associated with DCR (odds ratio, 0.09; 95% CI 0.01-0.83; *p*=0.034).

**Table 3:** Univariate and multivariate analyses of potential factors associated with disease control rate.

	No. of case	Univariate analysis		Multivariate analysis	
		ORs (95% CI)	*p* values	ORs (95% CI)	*p* values
Sex					
Male	31	0.44	0.344		
Female	21	(0.08-2.42)			
Age				0.17	
≥65 years	32	0.19	0.133	(0.02-1.79)	0.139
<65 years	20	(0.02-1.66)			
Body weight					
≥65 kg	18	1.71	0.538		
<65 kg	34	(0.31-9.52)			
Smoking history					
Never	23	1.39	0.678		
Current/former	29	(0.30-6.54)			
ECOG PS					
≤1	43	1.76	0.536		
≥2	9	(0.29-10.58)			
Dosage					
≥10mg	39	0.26	0.089	0.3	0.194
8mg	13	(0.05-1.23)		(0.05-1.85)	
ANL C_trough_					
≥10.29ng/ml	32	0.07	0.02	0.09	0.034
<10.29ng/ml	20	(0.01-0.69)		(0.01-0.83)	
Histology					
Adenocarcinoma	31	0.87	0.857		
Squamous	21	(0.18-4.09)			
TNM stage					
III stage	18	0.86	0.862		
IV stage	34	(0.18-4.11)			
Driver alterations					
No	37	1.6	0.559		
Yes	15	(0.33-7.55)			
AEs: Hypertension					
Yes	27	1.32	0.721		
No	25	(0.29-5.95)			
AEs: Hand-foot syndrome	29	2.41	0.267		
Yes	23	(0.51-11.37)			
No					
AEs: Thyroid stimulating hormone elevation					
Yes	21	1.39	0.678		
No	31	(0.30-6.54)			
AEs: Hypertriglyceridemia					
Yes	22	1.27	0.765		
No	30	(0.27-5.97)			

ANL, anlotinib; AEs, adverse events; ORs, odds ratio. ECOG, Eastern Cooperative Oncology Group; PS, performance status; TNM, tumor node metastasis.

### Association of toxicity with ANL C_trough_

**Figure 4:**
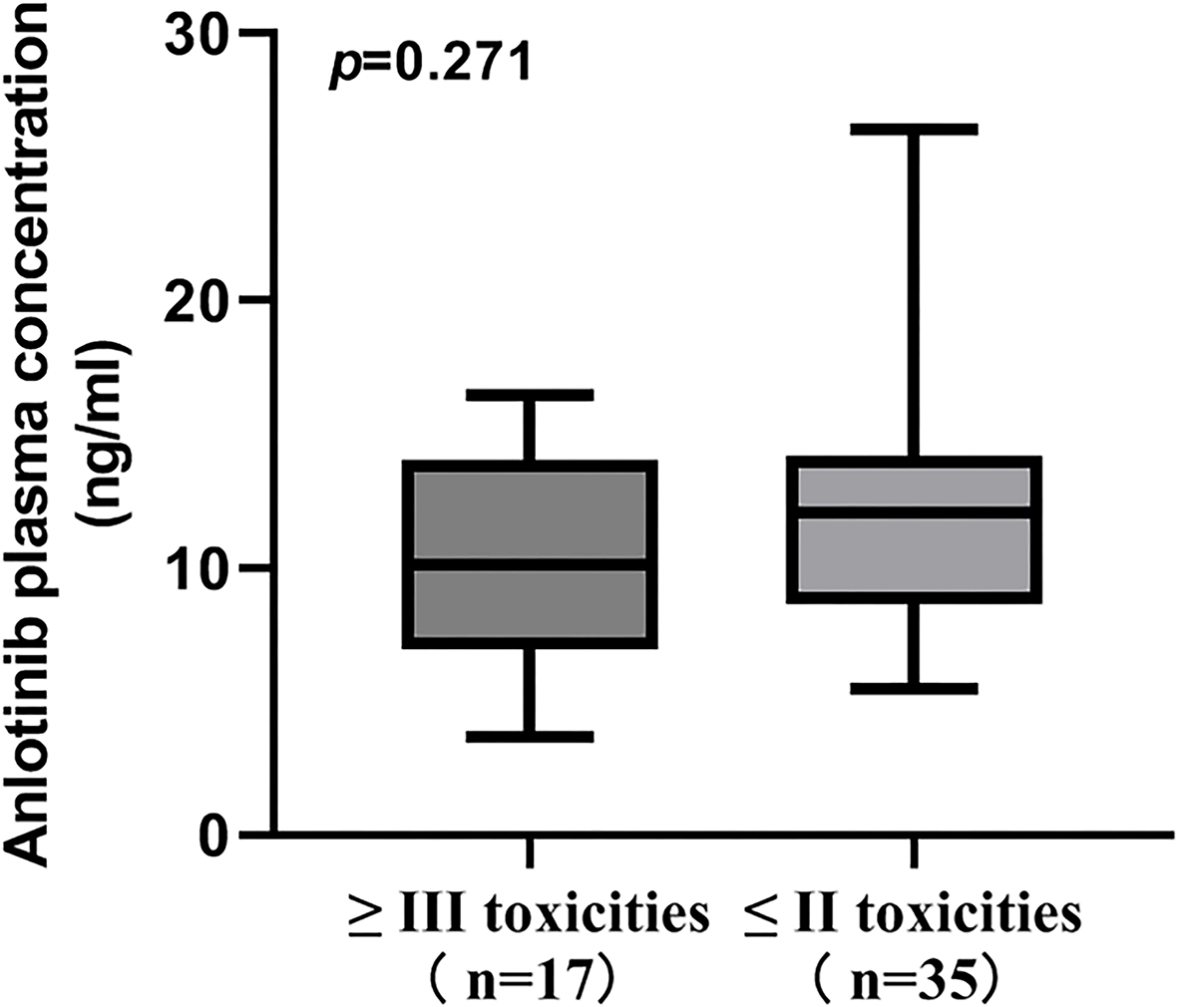
Comparison of ANL C_trough_ between patients with grade ≥ III toxicities and grade ≤ II toxicities.

In the present study, the most common toxicities were hypertension, hand-foot syndrome, hypertriglyceridemia and thyroid-stimulating hormone elevation. Of these, the most common grade ≥3 toxicities were hypertension (21.15%), hand-foot syndrome (3.85%) and thyroid-stimulating hormone elevation (3.85%). No significant difference in ANL C_trough_ was found when comparing grade ≥ 3 toxicities with grade ≤2 toxicities (*p*=0.271), which were depicted in **Figure 4**. All patients in the high-exposure group and low-exposure group experienced toxicities induced by ANL in **Table 4**. Although there was no significant difference in the incidence of most toxicities among high-exposure group and low-exposure group, the incidence of any grade hand-foot syndrome (70.00% vs 36.36%, *p*=0.016) and any grade thyroid-stimulating hormone elevation (53.33% vs 22.73%, *p*=0.026) was significantly higher in the high-exposure group than the low-exposure group.

**Table 4:** Comparison of adverse events in patients with ANL C_trough_ ≥10.29ng/ml (high-exposure group) and <10.29ng/ml (low-exposure group).

Type	Level	high-exposure group	low-exposure group	*p* values
		(n=30) (%)	(n=22) (%)	
Hypertension	Any grade	16 (53.33)	11 (50.0)	0.812
	≥ Grade III	5 (16.67%)	6 (27.28)	0.355
Hypertriglyceridemia	Any grade	12 (40.0)	10 (45.45)	0.694
	≥ Grade III	1 (3.33)	1 (4.55)	0.822
Hand-foot syndrome	Any grade	21 (70.0)	8 (36.36)	0.016
	≥ Grade III	2 (6.67)	0 (0)	0.217
Diarrhea	Any grade	6 (20.0)	6 (27.27)	0.539
	≥ Grade III	0 (0)	1 (4.55)	0.238
Thrombocytopenia	Any grade	3 (10.0)	2 (9.09)	0.972
	≥ Grade III	1 (3.33)	0 (0)	0.387
Fatigue	Any grade	8 (26.67)	6 (27.27)	0.961
	≥ Grade III	0 (0)	1 (4.55)	0.238
Proteinuria	Any grade	5 (16.67)	6 (27.27)	0.355
	≥ Grade III	1 (3.33)	0 (0)	0.387
Hemoptysis	Any grade	6 (20.0)	3 (13.64)	0.549
	≥ Grade III	1 (3.33)	0 (0)	0.387
Thyroid-stimulating hormone elevation	Any grade	16 (53.33)	5 (22.73)	**0.026**
	≥ Grade III	2 (6.67)	0 (0)	0.217

Bold values indicate that the difference were statistically significant.

## Discussion

In the present study, the median ANL C_trough_ was 11.45ng/ml (range, 3.69-26.36 ng/ml) and shows large inter-patient variability with 37.66% CV, which was similar to the previous studies, and ANL occasionally cause no response in patients with NSCLC (9–11). The influence of plasma concentration on clinical response should be considered during ANL therapy. A preclinical study showed that ANL have high selectivity for vascular endothelial growth factor (VEGF) family members, especially VEGFR2, with half maximal inhibitory concentration (IC50) value was 0.2 ng/ml in endothelial cell (2b). However, information on the association between ANL exposure and treatment outcomes is limited. To our knowledge, this study was the first reported that ANL C_trough_ was higher in the PR group than in the PD groups, and the threshold value of C_trough_ associated with the responder for advanced NSCLC was 10.29 ng/ml using ROC analysis combined with Youden index. In the non-responder group (n=8), ANL C_trough_ of 87.5% (7/8) non-responder patients were below the 10.29ng/ml, which means that threshold value (10.29ng/ml) could predict non-responder in advanced NSCLC treated with ANL. Furthermore, ANL C_trough_ show partially dose-dependent and there was significant difference between the high-exposure group and low-exposure group at a dosage of 8mg versus ≥10mg of ANL per day, which is consistent with the *Tan T et al.* study (15). ANL C_trough_ ≥ 10.29ng/ml was achieved by changing the dose administered, which might obtain sufficient efficacy. *Yu M et al.* and *Li Z et al.* have been reported that the plasma concentration of ANL in female was higher than male patients, but this study found that gender could not affect the ANL C_trough_ (14, 16). Dexamethasone and CYP2C19 gene polymorphisms (rs3814637 and rs11568732) may also influence the ANL exposure (14, 15). However, we did not found significance interaction of ANL with other drugs, which were shown in **Table 2**. Subsequent studies could focus on the influence of gene polymorphisms, drug interactions and other factors on ANL C_trough_.

In this study, the DCR was 84.6% (44/52), which was similar to the ALTER 0302 (DCR 83.3%) and ALTER 0303 study (DCR 81.0%) (Han and Li and 6, 21). However, this study divided the patients into the high-exposure group (≥10.29ng/ml) and low-exposure group (<10.29ng/ml) according to the threshold value. The DCR can reach 96.67% (29/30) in the high-exposure group, which was higher than 83.3% and 81.0%. *Wang J et al.* reported that ANL-induced hypertension, hand-foot syndrome, hypertriglyceridemia and elevation thyroid stimulating hormone were independent predictor factors of PFS in refractory NSCLC treated with ANL(6). However, our present study has not investigated the association between the ANL-induced hypertension, hand-foot syndrome, hypertriglyceridemia and elevation thyroid stimulating hormone and DCR, which were shown in **Table 3**. Multivariate logistic regression indicated that ANL C_trough_ ≥10.29ng/ml was an independent prognostic factor with DCR in this study. It is possible that PFS and DCR are different efficacy evaluation indicators. According to the results of this study, we proposed that maintaining ANL C_trough_ ≥10.29ng/ml is crucial for achieving the DCR.

In clinical practice, toxicities often lead to treatment discontinuation or dose reductions. The most common toxicities associated with ANL were hypertension, hand-foot syndrome, hypertriglyceridemia and thyroid-stimulating hormone elevation in this study. Notably, hypertension was the most common adverse events during ANL treatment, which is consistent with the ALTER 0302 and ALTER 0303 (21, 21). According to the ALTER 0303, most of toxicities occurred within the first two cycles of ANL treatment, the follow-up period of toxicities was also set the first two cycles in this study. Although there was no significantly difference with grade ≥ 3 toxicities and grade ≤ 2 toxicities between the high-exposure group and low-exposure group, the incidence of any grade hand-foot syndrome and thyroid-stimulating hormone elevation was significantly higher in the high-exposure group in this study. Based on the exploratory data from a phase I trial, ANL exhibited a quite long t_1/2_ (96 ± 17h) and accumulation of plasma over time with accumulation ratio of 12, suggesting that drug accumulation over time (8). *Zhang J et al.* has been reported that the initial dosage was 12mg ANL in a patient, C_trough_ level in cycle 1 and 2 of treatment was 12.75 and 15.83ng/ml, respectively. However, C_trough_ level increased to 44.01 ng/ml in cycle 4 with intolerance to toxicity, then the dose was reduced to 10mg from cycle 5. Thereafter, C_trough_ level decreased to 20.84 ng/ml (22). The association between ANL C_trough_ and toxicities was not examined at long-term in the present study. Considering the accumulation effect of ANL, it is necessary to explore the association between the ANL C_trough_ and toxicities by long-term follow-up period.

There were some limitations to the present study. First, we were unable to estimate the area under the plasma concentration-time curve of ANL, which is an indicator of the drug exposure *in vivo*. However, for molecularly targeted drugs, such as sunitinib, imatinib and pazopanib, TDM based on C_trough_ level is recommended (11, 23). Second, this study was a single-center study. The number of patients involved was small and the follow-up period was short. For confirming these findings, multiple-center study and large PK data should be performed by long-term follow-up. Third, this study could not evaluate the association between the ANL C_trough_ and PFS or OS. The contribution of ANL C_trough_ to PFS and OS to be further defined.

In conclusion, this is the first study to elucidate the relationship between ANL C_trough_ and clinical efficacy and toxicities in Chinese patients with advanced NSCLC. The present study demonstrated that there was an extensive inter-individual variability in ANL C_trough_, and maintaining C_trough_ ≥10.29ng/ml is crucial for achieving the responder in advanced Chinese NSCLC patients treated with ANL. The ANL C_trough_ could be used as a guide to improving efficiency.

## Data availability statement

The original contributions presented in the study are included in the article/**Supplementary Material**. Further inquiries can be directed to the corresponding author.

## Ethics statement

The studies involving human participants were reviewed and approved by the Ethics Committee of the Hangzhou First People’s Hospital (Ethical approval documents: No.2020-098-01). The patients/participants provided their written informed consent to participate in this study.

## Author contributions

LJ and LC conceptualized and supervised the project. L-SW and JY carried out the study and interpreted the results. HJ drafted the initial version of the manuscript. WY and J-JR searched the literature and collected data. All authors contributed to the article and approved the submitted version.
