# Supplementary figures and images for: Association between anlotinib trough plasma concentration and treatment outcomes in advanced non-small-cell lung cancer
Source: Front Oncol. 2023 Mar 3;13:1146362. doi: 10.3389/fonc.2023.1146362 (PMC10020721; doi:10.3389/fonc.2023.1146362)

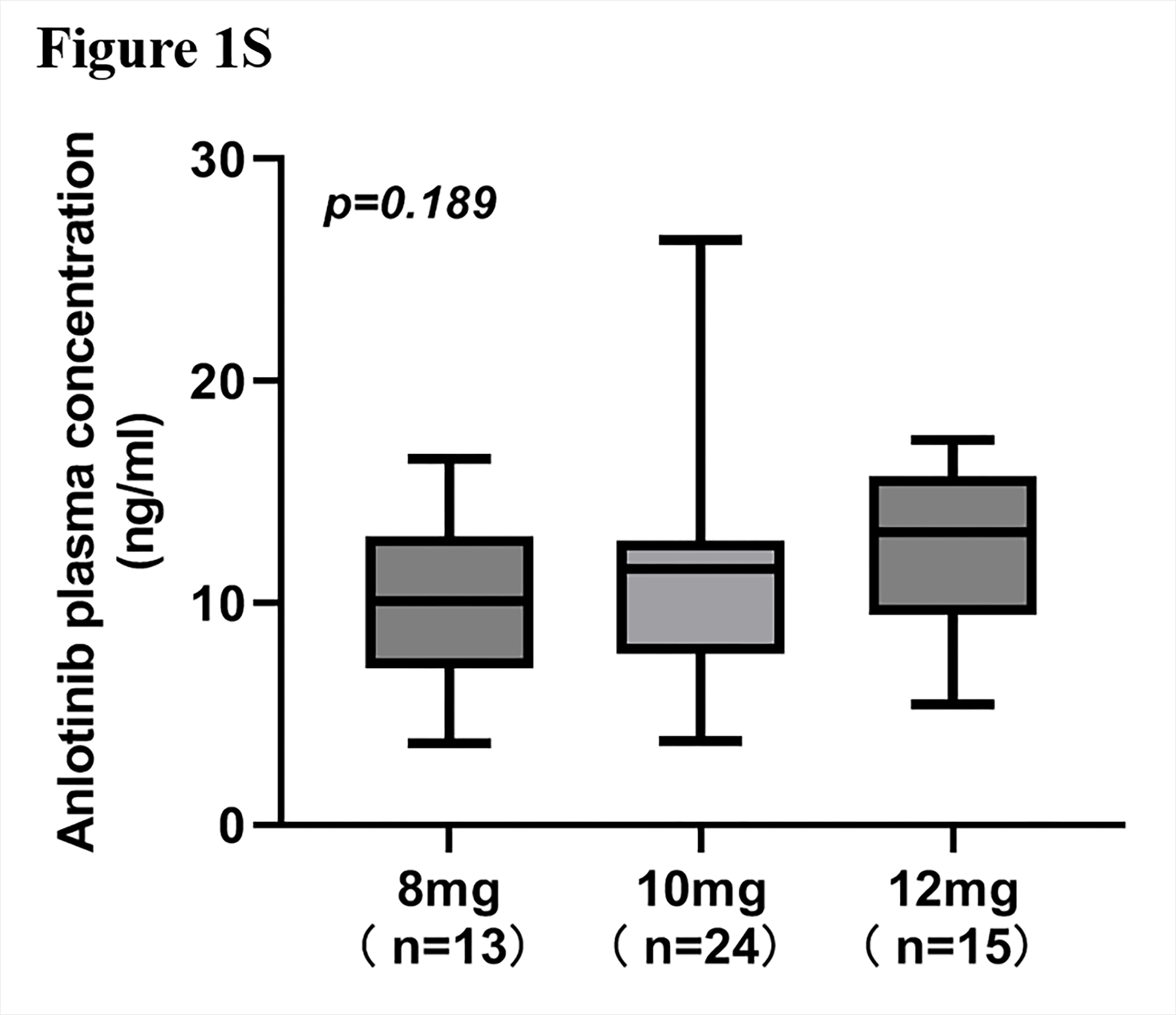

Supplement: Supplementary file 1 [file Image_1.tif]

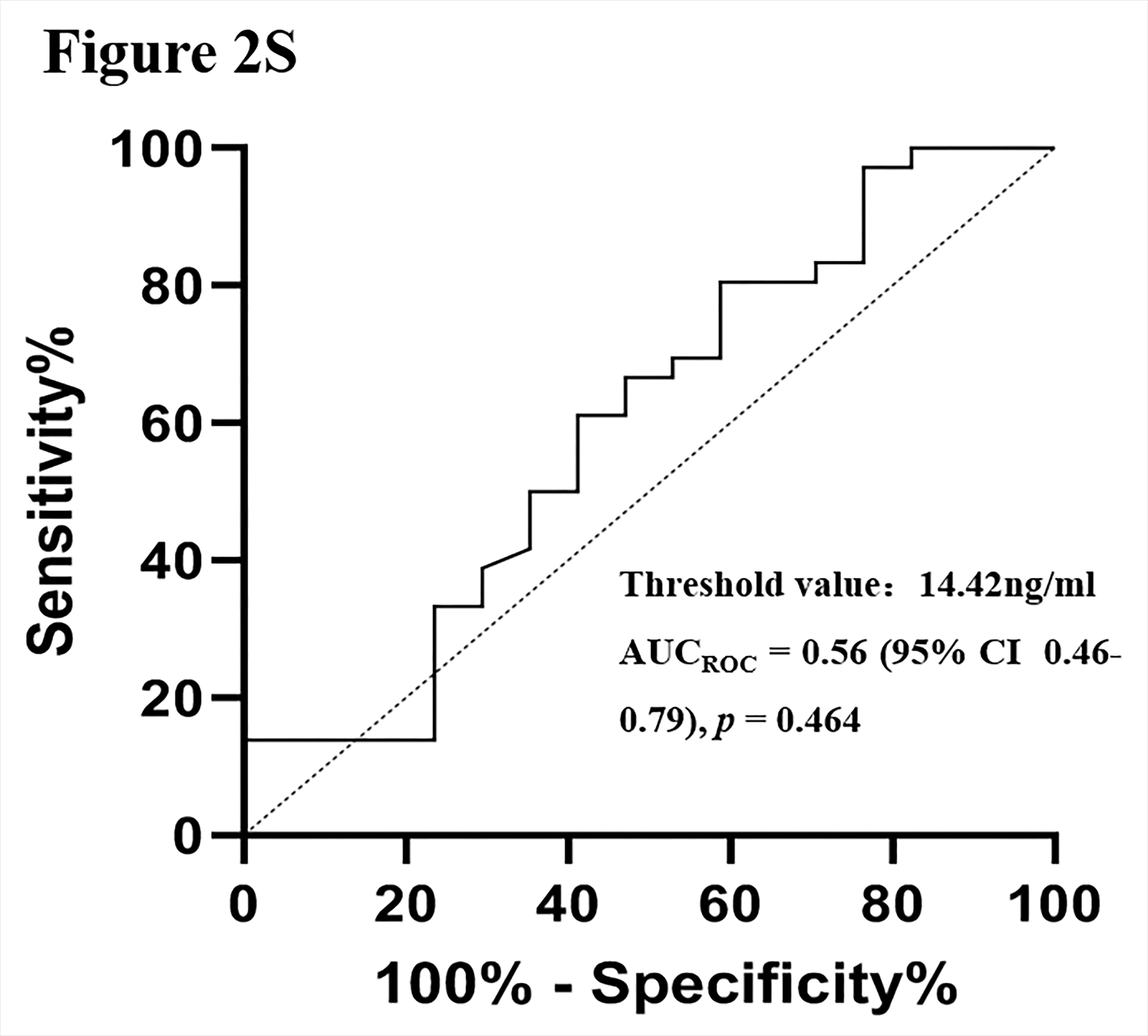

Supplement: Supplementary file 2 [file Image_2.tif]
